# The unassailable nature of ground truth in scientific research: Response to Asonov et al.

**DOI:** 10.1016/j.fsisyn.2024.100556

**Published:** 2024-09-16

**Authors:** Kyriakos N. Kotsoglou, Alex Biedermann

**Affiliations:** Northumbria University, Northumbria Law School, Newcastle upon Tyne, UK; University of Lausanne, Faculty of Law, Criminal Justice and Public Administration, School of Criminal Justice, 1015 Lausanne–Dorigny, Switzerland

Dear Editor,

In our recent article *Polygraph-based deception detection and Machine Learning. Combining the Worst of Both Worlds?* [1], we critically exposed the drawbacks of the tendency to apply machine learning (ML) methods to ad hoc convenience data. To illustrate our arguments, we referred to a recent publication on polygraph-based deception detection [2]. Our main argument was that training ML models on data with human-assigned labels, rather than actual ground truth, does not meet the requirements for developing and validating evidence evaluation systems currently used in several areas of forensic science, such as fingermark examination [3] and automated human-supervised forensic voice comparison [4]. The requirement for known ground truth data is also emphasized for AI-based methods for use in legal systems more generally [5]. In a rejoinder to our article, Asonov et al. [6] make a number of claims to which we respond below.

## 1 On the training on and the correction of “dirty labels”

Asonov et al. state that the “[m]ajor critique (…) by Kotsoglou et al. (…) is that we [Asonov et al.] train on dirty (non-ground truth) labels” [6, p.1]. Asonov et al. respond that they inspected their field training data and “(…) found 30 problematic examiner conclusions” [6, p.1], and “(…) then trained the production model with these 30 errors corrected” [6, p.1].

This does not address our criticism. Our point is that the “historical data of 2094 field polygraph screening recordings” [2, p.2] have human-assigned labels, which means that the actual ground truth in each of those cases is, by definition, unknown. Inspecting such data, sorting out a certain number of “problematic examiner conclusions” [6, p.1], having them “reviewed” by human examiners, and eventually changing their labels does not fix the problem: they still have human-assigned labels.

Moreover, the attempt to assess the (diagnostic) performance of a method by suspending the very idea of ground truth is awry. The approach of Asonov et al. is indefensible not only on methodological grounds. Legal orders around the world require that judges receive information about the error rate of a particular method (see the *Daubert* standard in the US, *Daubert v. Merrell Dow Pharmaceuticals Inc., 509 U S. 579 (1993)*^1^ and Privy Council in *Lundy v R* [2013] UKPC 28). It is doubtful how a purely self-referential system, which merely mimics imperfect human examiners and whose congruence with actual ground truth is *by design* unknown, could satisfy this requirement.

Furthermore, calling a change in labelling a “correction”, which suggests an alignment with ground truth is misleading because human reviewers are not clairvoyant. At best, such data could be called “adjusted”.

## 2 On the notion of error

Asonov et al. reaffirm that examiner “errors” can be detected “(…) when a professional, unbiased examiner reviews the screening” [6, p.1]. This is confusing, to say the least. What a reviewer could perhaps do is either *agree* or *disagree* with the opinion of a first examiner. But that is an act of will, not a cognitive, let alone scientific, enterprise. At best, if the reviewer has a special status, such as a senior examiner, or is considered some sort of reference point for whatever reason or qualification, then any disagreement with the first examiner's opinion is merely a mismatch with respect to the reference opinion. However, it is not an *error* because the reference opinion itself is just an opinion about ground truth.

## 3 The claim that polygraph-based deception detection is a “legitimate technique”

Asonov et al. state that “[p]olygraph-based deception detection is a *legitimate* technique in many countries, including in the UK” and that “[t]here is simply no need to legitimize it” [6, p.1]. This statement confuses the question of legitimacy with the question of the scientific status of polygraph-based deception detection. Formal authorization for the use of a particular method or technique, however widespread, is a mere regulatory matter and a *statement of fact*, which we do not dispute. No ‘Ought’ can be derived from a ‘Is’. Asonov et al. conflate the empirical issue (that there is a polygraph industry as a matter of fact; similarly: there is an astrology industry) and the normative aspect (that such fields have a questionable scientific status and should not become part of the arsenal of public authorities). Crucially, however, and more importantly, it is, to put it mildly, inaccurate, if not misleading, to say that ‘the use of the polygraph is a *legitimate* technique in the UK’. First, the UK (like the US) is not a single criminal justice jurisdiction. In England and Wales, the polygraph is used only in the context of probation. Second, with respect to polygraphic data and related information, Section 30(1) of the Offender Management Act 2007 provides that evidence of any matter mentioned during a polygraph session *may not* be used in any proceedings against the interviewee (i.e., the released offender) for an offence. The aforementioned matters could be “(a) any statement made by the released person while participating in a polygraph session; and (b) any physiological reactions of the released person while being questioned in the course of a polygraph examination” (Offender Management Act 2007, Section 30(2)).

Thus, it is the law itself that recognizes the lack of validity of polygraph-based interviews. Moreover, consumers of polygraph-based technology may have little regard for its actual truth-conduciveness as long as it serves as an interrogation tool that pushes examinees to confess.

Our point about legitimacy is a different one. We are concerned that the claim that the scientificity of polygraph-based deception detection could be improved by using ML as an “add-on” might inappropriately serve as an argument for the legitimacy of the procedure. However, as noted in point 1 above, training on data with human-assigned labels, *even when* reviewed and adjusted by human examiners, remains agnostic to the ground truth as the relevant reference point, and thus fails to meet an important requirement for legitimacy in legal proceedings.

## 4 On *Forensic Science International: Synergy* being a pseudoscientific journal

Asonov et al. [6] hypothesize that our description of polygraph-based deception detection as pseudoscience suggests that *Forensic Science International: Synergy* (FSI SYN) is a pseudoscientific journal because it has previously published a contribution on the polygraph. However, the contribution to which Asonov et al. [6] refer was not a regular submission. It is not even a paper, but a less than 300-word conference abstract of a case report [7]. It is part of the Proceedings of the American Society of Crime Laboratory Directors (ASCLD) Meeting 2023. The Proceedings of the ASCLD Meeting 2023 were published as an FSI SYN Supplement. In this case, FSI SYN was merely the publication channel. The decision to accept [7] was made by and was the responsibility of the respective conference organizers.

## 5 On the role of methodological critique

Asonov et al. state that we [Kotsoglou and Biedermann] “cannot or wish not [to] conduct research that minimizes deficiencies of the widespread, de-facto, and in many cases de-jure, standard of probabilistic deception detection (polygraph) employed by police, special government agencies, and the private sector. However, what is the point then in spending time criticizing those who do? Instead, wouldn’t it be more productive to research an alternative method free of deficiencies?” [[6], p. 2].

We understand that Asonov et al. are not academics, but industry-based, applied researchers. Nevertheless, they should know that addressing methodological issues in the scientific literature is part and parcel of scholarly work. Asonov et al. wonder what’s the point of critics wasting their time chasing a zombie,^2^ and whether it wouldn’t be more productive if we [Kotsoglou and Biedermann] tried to create our own zombie. The answer, of course, is *no*, because that would require us to start from questionable premises. As every blue-ribbon committee on (the validity of) polygraph-based deception detection has confirmed, the so-called stress response to be measured can be triggered by a variety of factors embedded in case-specific circumstances that are difficult to control, thus undermining the idea of deception detection based on physiological indicators.

Furthermore, it is unwarranted to speak of ‘*probabilistic* deception detection’. As we have repeatedly noted, the validation of probabilistic evaluation methods in forensic science requires known ground truth data sets [[3], [4], [5]], which are absent from Asonov et al.’s account.

## 6 On the use of polygraph-based deception detection as the sole source of information (“lie-detection”) and fallibility

Asonov et al. state that they “(…) agree that polygraph test conclusions simply cannot be used as a sole source of information in internal or criminal investigations, partially because the method is prone to errors (…).” [6, p.2]

This statement is a truism and a distraction. It is not disputed that one source of information is not usually used in isolation (i.e., as a “sole source”). However, this does not mean that a flawed method, such as polygraph-based deception detection, should be considered useable because there may be other sources of information in a case that could somehow “cover up” a misleading direction triggered by the flawed method.

We also disagree that one can meaningfully say that a given method is merely “prone to errors”. Fallibility is a fact of life. But the problem lies elsewhere. We all use fallible methods on a daily basis. The problem is that Asonov et al. turn a blind eye to the actual truth conduciveness of their method (polygraph-based deception detection). Instead, they turn it into a self-referential method by using human-assigned ground truth *opinions*, i.e. human beliefs, as unvalidated substitutes for actual ground truth, and then promote the method as fit for purpose. This difference in reference points is subtle, but paradigmatic. It touches on basic axioms of research, including centuries-old metaphysical substrate, i.e. that something can either be true or not, regardless of the difficulties of verifying which is the case.

Potential buyers of products that claim ML can (magically) fix inherent design flaws in polygraph-based deception methods should not ignore that the price they may be paying is the abandonment of fundamental principles. It is high time for all of us to seriously question not only whether we should buy the claims of some parts of the ML industry, but more importantly, whether such claims should be made in the first place.

We thank Asonov et al. for the constructive exchange.

## Declaration of competing interest

We declare that we have no known competing financial interests or personal relationships that could have appeared to influence the work reported in this paper.

## Footnotes

1 https://supreme.justia.com/cases/federal/us/509/579/.

2 See Kotsoglou [8] for why polygraph-based deception detection can be considered a forensic zombie technique.
